# Three‐dimensional endoanal ultrasound measurements for the assessment of perineal body size in a cohort of Swedish nulligravidae

**DOI:** 10.1111/aogs.70227

**Published:** 2026-06-17

**Authors:** Emilia Rotstein, Nina Rose, Marianne Starck, Hedvig Engberg

**Affiliations:** ^1^ Department of Clinical Science Intervention and Technology Karolinska Institutet Stockholm Sweden; ^2^ Department of Gynecology and Reproductive Medicine Karolinska University Hospital Stockholm Sweden; ^3^ Department of Surgery, Pelvic Floor Centre Skåne University Hospital Malmö Sweden; ^4^ Department of Women's and Children's Health Karolinska Institute Stockholm Sweden

**Keywords:** endoanal, nulliparity, pelvic floor anatomy, perineal body, perineum, ultrasonography

## Abstract

**Introduction:**

Despite the perineal body's important functional role, few studies have specifically evaluated its size in women. Existing three‐dimensional ultrasound studies typically involve small samples, often including both nulliparous and parous women, and focus on measuring internal and external sphincter thickness rather than the perineal body itself.

**Material and Methods:**

This cross‐sectional study primarily aimed to measure the size of the perineal body in a population of nulligravidae (*n* = 148) using three‐dimensional endoanal ultrasonography. Secondary aims were to (1) evaluate the feasibility and reproducibility of 3D ultrasound interpretation across raters with varying levels of experience and (2) investigate how perineal body measurements correlated with body composition.

**Results:**

The study participants had a mean age of 31 years (SD ± 6), a mean height of 167 cm (SD ± 5.9), and a mean body mass index (BMI) of 25.3 kg/m^2^ (SD ± 5.6). The perineal body had a mean proximal–distal length of 17.5 mm (SD ± 3.0), with excellent interrater reliability (ICC = 0.89; 95% CI: 0.85–0.92). The mean antero–posterior height was 9.7 mm (SD ± 1.4; ICC = 0.67, 95% CI: 0.57–0.75), and the mean perineal body area was 120 mm^2^ (SD ± 28; ICC = 0.84, 95% CI: 0.77–0.88). Results suggest that perineal body measurements obtained through standardized 3D endoanal ultrasonography are reproducible across raters, supporting the method's feasibility regardless of prior ultrasound experience. BMI and weight showed a moderate positive correlation with both the length and the area of the perineal body (*p* = 0.05).

**Conclusions:**

Standardized 3D endoanal ultrasonography provides reproducible measurements of the perineal body in nulligravid women and measurements correlated moderately with body composition. This suggests the use of this imaging method in asymptomatic populations and future research on pelvic floor function.

## Graphical abstract

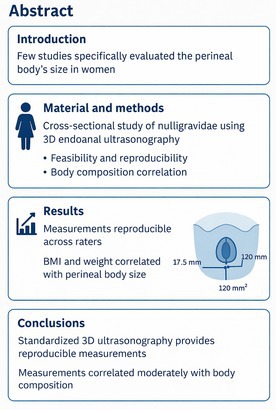

**Abbreviations**
3D: three‐dimensional
BMI: body mass index
EAUS: endoanal ultrasound
KAPTAIN: Karolinska symptoms after perineal tear inventory

**Key message**

We provide normative data on perineal body dimensions in nulliparous women using high‐resolution 3D ultrasound. These findings enhance anatomical understanding and may support clinical assessment of perineal integrity in women.

## 1 INTRODUCTION

The female pelvic floor is comprised of a complex interaction of bone, muscles, connective‐ and supportive tissues, harboring the pelvic organs.^1^ It is defined by two muscular layers: the proximal levator ani plane and the distal plane of the perineal body. The perineal body is the most distal fibromuscular structure of the pelvic floor, adhering to the muscularis layer of the vagina and the perineal membrane anteriorly; the posterior attachment is formed by the rectovaginal fascia, the distal insertion of the levator ani muscle and the external anal sphincter. It is imperative to pelvic organ support by maintaining the integrity and morphology of the genital hiatus.^2^ The most common cause of injury to the perineal body is obstetric trauma and is estimated to occur in approximately 80% of vaginal deliveries.^3^ Consequently, the support of the pelvic organs and functional anatomy of the pelvic floor lean heavily on an intact perineal body.^4^, ^5^

Increased knowledge of perineal body dimensions is essential for understanding its role in maintaining the structural and functional stability of the posterior compartment and in the development of, for example, procto‐/rectocele or anorectal dysfunction.^6^ There is still an ongoing debate regarding its attachments to the distal levator ani muscles and the supportive tissue of the pelvic floor.^7^, ^8^, ^9^ Whether using a two‐, three‐, or four‐dimensional approach, ultrasound is a valuable clinical tool for visualizing the intricate pelvic floor structures, as it is accessible and highly tolerable.^10^, ^11^, ^12^, ^13^, ^14^ Three‐dimensional ultrasound adds the benefits of multiplane imaging and the reliability of 3D endoanal ultrasound (3D‐EAUS) has already been validated by previous studies.^15^, ^16^

Studying nulliparous women allows for characterization of the size of the perineal body in its likely unaffected state, providing a reference point for future comparative studies. Given the limited evidence regarding the perineal body in nulliparous women, the aim of this study was to describe its measurements in a fertile, nulliparous population. We further hypothesized that perineal body size would correlate with body constitution, specifically height, weight, and BMI. Secondary aims of the study were to evaluate whether standardized 3D‐EAUS measurements of the perineal body could be reliably performed and interpreted by raters regardless of prior ultrasound experience.

## 2 MATERIAL AND METHODS

### 2.1 Study design and sample

This is a cross‐sectional study of fertile nulliparas that were recruited between September 2018 and February 2020. Study participants were approached through study information leaflets posted at the Department of Gynecology and Reproductive medicine at Karolinska University Hospital, informational talks for medical and midwifery students and through posts with study information on social media. Inclusion criteria were fertile nulliparous women with Swedish proficiency, and exclusion criteria were prior anorectal surgery.

### 2.2 Data collection

Data were pseudonymized and the code key was securely stored and accessible only to authorized members of the research group.

Demographic data such as age, height, weight, tobacco use, and medical history were collected using a study‐specific questionnaire.

### 2.3 Three‐dimensional endoanal ultrasound (3D‐EAUS) technique

The participants were examined in a dorsal lithotomy position with an emptied bladder and no prior bowel preparation. Care was taken to align the probe with the axis of the anal canal, adding no extra pressure to the perineum. All exams were carried out by one of the investigators (NR).

The 3D‐EAUS examination was performed with a BKMedical Flex Focus equipped with a high‐resolution 8838 probe. It has a built‐in automatic 6 cm linear array that rotates 360° and acquires 1440 two‐dimensional images of 0.25° each in 67.9 s. A setup of 12 K MHz and a focal range of 35 mm allows acquisition without any movement of the probe. The volumes were rendered during rest and stored digitally for later offline analysis.

### 2.4 Ultrasound volume analysis

**FIGURE 1:**
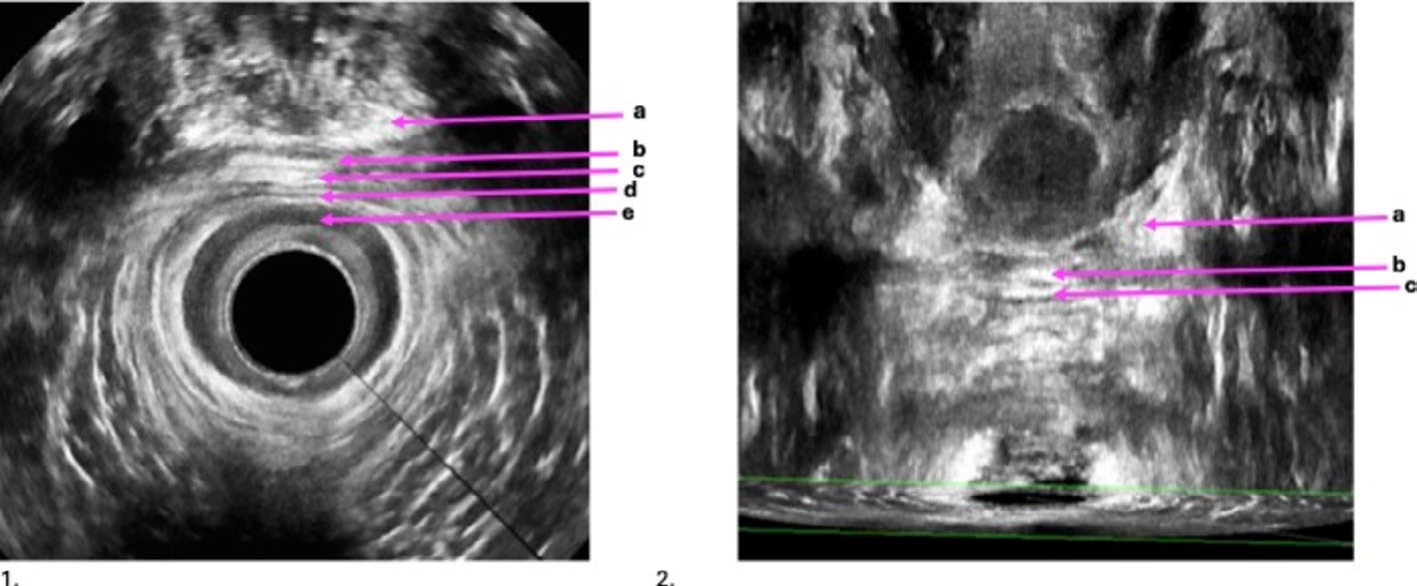
Axial (1) and coronal (2) view of the perineal muscles. Individual muscles marked: (a) puboperineal insertion, (b) deep transverse perineal muscle, (c) superficial transverse perineal muscle, (d) external anal sphincter, and (e) internal anal sphincter.

After review of existing literature, a standardized protocol for analysis of the rendered volumes was developed, *based on previously published anatomical definitions and measurement approaches for the perineal body in pelvic imaging*.^7^, ^13^, ^14^, ^17^, ^18^, ^19^ Measurements were performed in the axial, sagittal, and coronal planes. Each 3D‐EAUS volume was first marked at 12 o'clock in the axial plane, corresponding to the site where the puboperineal muscles attach to the transverse perineal muscle. When assessing the puboperineal insertion, both the left and right pedicles and their attachments to the transverse perineal muscle were identified (Figure 1).

**FIGURE 2:**
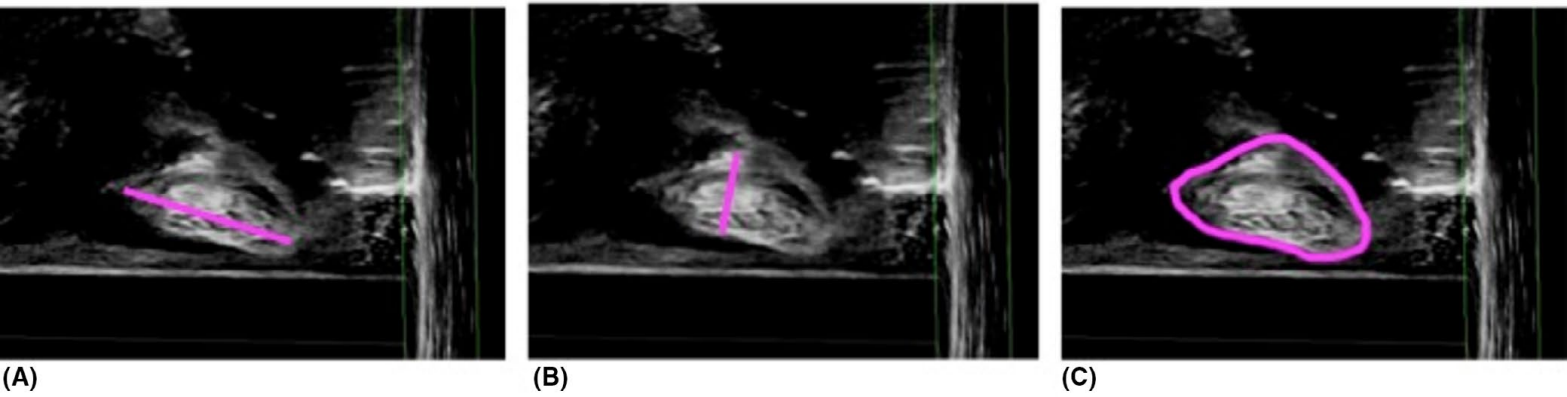
Measurements of the proximodistal (A) and anterior–posterior (B) distances in the sagittal view, as well as the area (C).

The ultrasound volume was then rotated to the sagittal plane, where the length, height, and area of the perineal body were measured in the midline. Length was defined as the longest distance in the proximal–distal direction, and height was measured perpendicularly in the cranio‐caudal direction at the thickest point in the anterior–posterior dimension. Area was measured by tracing the entire visible contour of the perineal body in the midline (Figure 2). Although 3D ultrasound volumes were acquired, the anatomical volume of the perineal body could not be measured, as the lateral borders extend into the transverse perineal muscles and cannot be clearly distinguishable on ultrasound.^13^ As a result, only area measurements were performed, and anatomical volume estimation was not feasible. To confirm the study protocol, 20 volumes were assessed independently by three of the authors (NR, ER, MS), and results were compared to reach a consensus on measurement definitions and identification of anatomical landmarks. *This process aimed to ensure methodological consistency rather than to formally assess inter‐rater reliability*. All remaining 3D ultrasound volumes were assessed independently by the novice rater (NR) and one experienced rater (MS).

### 2.5 Statistical analyses

Data were analyzed using SPSS Statistics for Windows, version 22.0 (IBM Corp., Armonk, NY). Descriptive data and measurements are presented as means with standard deviations (SD) or median with interquartile range (IQR). Since the mean and median values were closely aligned for the anatomical measurements, indicating an approximately symmetric distribution of the data, the mean was used as the primary measure of central tendency in the results. A *p* <0.05 was considered statistically significant.

Inter‐rater reliability between the novice and experienced rater was evaluated using intraclass correlation coefficients (ICC) based on a two‐way mixed effects model with absolute agreement. Pearson's correlation coefficient was calculated to assess associations between average mean perineal body measurements and participants' BMI, height, and weight.

## 3 RESULTS

A total of 169 participants were recruited and underwent 3D‐EAUS examination at the Department of Gynecology, Karolinska University Hospital. Seventeen (10%) ultrasound volumes were excluded due to poor imaging quality in the subsequent offline analysis, and four participants did not meet the inclusion criteria due to postmenopausal status. Thus, 148 participants (87.6%) were included in the final analyses. Background demographics are presented in Table 1. Regarding medical history, only five participants reported comorbidities that could potentially affect the reported outcomes: three with irritable bowel syndrome (IBS), one with hemorrhoids, and one with Crohn's disease with perianal involvement.

**TABLE 1:** Background demographics.

Variables	*n*	Value
Age (years), mean ± SD (range)	148	31 ± 6.0 (19–50)
Height (cm), mean ± SD (range)	148	167 ± 5.9 (154–183)
Weight (kg), mean ± SD	147	70.9 ± 17.3
BMI (kg/m^2^), mean ± SD	148	25.3 ± 5.6
Smoking, % (*n*)	148	4 (6)
Snuff, % (*n*)	148	8 (12)

The anatomical measurements of the individual raters are presented in Table 2.

**TABLE 2:** Perineal body measures divided by raters including intraclass correlation coefficient (ICC).

	Rater 1	Rater 2	Mean difference (mm)	Average mean (mm) (Rater 1 + 2)	Intraclass correlation coefficient (95% CI)
Proximodistal distance (mm), mean (SD)	17.7 (3.0)	17.3 (3.1)	0.4	17.5	0.89 (0.85–0.92)
Anteroposterior distance (mm), mean (SD)	9.6 (1.5)	9.8 (1.6)	−0.2	9.7	0.67 (0.57–0.75)
Area (mm^2^), mean (SD)	122 (28)	118 (29)	4	120	0.84 (0.77–0.88)

Correlation coefficients were interpreted according to conventional thresholds: 0–0.19 = very weak, 0.20–0.39 = weak, 0.40–0.59 = moderate, 0.60–0.79 = strong, and 0.80–1.0 = very strong correlation, adapted from Mukaka.^20^

The intraclass correlation coefficient (ICC) indicated excellent agreement for proximodistal distance, good agreement for area, and moderate reliability for anteroposterior distance. All ICC values were statistically significant (*p* < 0.05), supporting the reproducibility of standardized perineal body measurements using 3D‐EAUS across raters with different levels of ultrasound experience. The mean values used henceforth are the average mean for each participant as small absolute differences suggest good agreement between raters and to minimize rater bias.

Weight and BMI showed consistent and moderately strong positive correlations with the proximodistal distance and area of the perineal body, while height showed weaker associations. Anteroposterior distance correlated significantly with weight and BMI, but not with height (Table 3).

**TABLE 3:** Correlations of height, weight, and BMI of the women to perineal body measurements (average mean).

	Height *r* (*p*‐value)	Weight *r* (*p*‐value)	BMI *r* (*p*‐value)
Proximodistal distance (mm)	0.21 (0.012)	0.46 (<0.001)	0.41 (<0.001)
Anteroposterior distance (mm)	0.14 (0.101)	0.25 (0.002)	0.21 (0.009)
Area (mm^2^)	0.20 (0.013)	0.46 (<0.001)	0.41 (<0.001)

## 4 DISCUSSION

The primary aim of this study was to characterize the anatomy and dimensions of the perineal body in a large cohort of nulliparous, fertile women using high‐resolution 3D‐EAUS. Our findings establish normative reference values for perineal body size in this population, contributing to a better understanding of normal perineal anatomy. These values may assist clinicians in assessing whether the perineal body appears shortened or small in individual patients.

The inter‐rater reliability analyses showed excellent agreement for perineal body length and good agreement for area, while height demonstrated moderate reliability. The lower ICC for height may reflect greater variation in identifying the cranio‐caudal extent of the perineal body in the sagittal plane, particularly given the lack of clearly defined anatomical landmarks superiorly and inferiorly. In contrast, length and area are more easily delineated based on consistent proximal–distal and anterior–posterior boundaries. These findings suggest that, although 3D‐EAUS allows for standardized measurement of perineal body dimensions, certain parameters such as height may be more susceptible to inter‐rater variability—especially when raters differ in ultrasound experience. Nonetheless, the overall reliability across all parameters was acceptable, supporting the feasibility of using standardized 3D‐EAUS measurements of the perineal body in both clinical and research settings, regardless of prior ultrasound experience.

As a secondary aim, we explored associations between perineal body dimensions and anthropometric variables. These findings suggest that larger body size, particularly in terms of weight and BMI, is associated with increased perineal body dimensions. However, the magnitude of these associations was small which might this suggest that although body constitution may influence perineal anatomy to some extent, these differences are likely to be of limited clinical relevance.

The findings from this study support previous literature demonstrating that 3D‐EAUS is a reliable tool for visualizing perineal anatomy. In 2016, Santoro et al. reported on the bipartite structure of the perineal body and validated 3D endovaginal ultrasound as a highly accurate and reproducible modality.^12^ Their cohort, which included five cadavers and 44 asymptomatic women, showed perineal body dimensions similar to those observed in this study.

Other studies have examined perineal body size in different populations and using various imaging modalities. For instance, Oberwalder et al. used 3D endoanal ultrasound in parous women with fecal incontinence and found that a perineal body thickness ≥ 12 mm was generally not associated with sphincter defects unless prior reconstructive surgery had been performed.^10^ Similarly, MRI studies comparing women with and without pelvic organ prolapse have shown larger perineal bodies in women without prolapse, suggesting a possible protective role of perineal body size. Zhou et al. measured the perineal body in both nulliparous and parous women using transperineal ultrasound and reported values comparable to ours, despite differences in technique and anatomical landmarks.^14^ These converging results across studies support the robustness of the measurements in this study and reinforce the relevance of 3D‐EAUS in perineal assessment.

The strengths of this study are the number of participants, the structured protocol for analysis of the ultrasound volumes and the correlation with participants' body constitution. Even though there were two raters with substantially different levels of experience in assessing 3D‐EAUS volumes, the difference in measurement was small in absolute numbers. Clinically, they cannot be considered relevant even though statistically so. This may indicate that the protocol and measurement methodology are feasible and reproducible even by an inexperienced rater.

There is a limitation in that height and weight and thus BMI were self‐reported, which is a possible uncertainty. Socioeconomic background and level of education were not reported which might indicate a screwed sample selection, which might also be suggested by the low mean BMI. One final limitation is that the data presented is not correlated with clinical perineal body measurement or other imaging techniques. Asfour et al. have shown that 2D ultrasound can reliably quantify the perineal body and that these measurements correlate with posterior compartment prolapse, whereas POP‐Q PB does not accurately reflect perineal body dimensions. Their reported values are in good agreement with our own findings supporting the relevance of imaging‐based assessment.

## 5 CONCLUSION

This study provides normative data on perineal body dimensions in nulligravid women using standardized three‐dimensional endoanal ultrasonography. The method demonstrated good reproducibility across raters with different levels of ultrasound experience, supporting its feasibility in both clinical and research settings. Perineal body measurements showed moderate associations with body composition. Together, these findings contribute to a better understanding of perineal size in a healthy, nulliparous population, and may serve as a reference for future studies on pelvic floor dysfunction.

## AUTHOR CONTRIBUTIONS

**Emilia Rotstein:** Conceptualization, data curation, formal analysis, funding acquisition, methodology, project administration, resources, supervision, validation, visualization, and writing – original draft. **Nina Rose:** Data curation, formal analysis, investigation, methodology, project administration, resources, visualization, and writing – review and editing. **Marianne Starck:** Conceptualization, formal analysis, investigation, methodology, supervision, and writing – review and editing. **Hedvig Engberg:** Conceptualization, formal analysis, methodology, project administration, resources, supervision, validation, visualization, and writing – review and editing.

## FUNDING INFORMATION

No specific funding was obtained.

## CONFLICT OF INTEREST STATEMENT

There are no conflicts of interest for any of the authors.

## ETHICS STATEMENT

The study was approved by the Stockholm Ethical Review Board (Dnr 2017/1418‐31, October 11, 2017). Written and oral informed consent was obtained from all participants. Participants were informed that they could withdraw from the study at any given time.

## Data Availability

The data that support the findings of this study are available on request from the corresponding author. The data are not publicly available due to privacy or ethical restrictions.

## References

[1] Ashton‐Miller JA , JO DL . Functional anatomy of the female pelvic floor. Ann N Y Acad Sci. 2007;1101:266‐296. 17416924 10.1196/annals.1389.034

[2] Woodman PJ , Graney DO . Anatomy and physiology of the female perineal body with relevance to obstetrical injury and repair. Clin Anat. 2002;15:321‐334. 12203375 10.1002/ca.10034

[3] Edqvist M , Hildingsson I , Mollberg M , Lundgren I , Lindgren H . Midwives' Management during the second stage of labor in relation to second‐degree tears‐an experimental study. Birth. 2017;44:86‐94. 27859542 10.1111/birt.12267 PMC5324579

[4] Sultan AH , Thakar R , Fenner DE . Perineal and Anal Sphincter Trauma ‐ Diagnosis and Clinical Management. Springer London; 2007.

[5] Caffee HH . Elective perineorrhaphy in the delivery room. Bull Dade Cty Med Assoc Inc. 1947;17:29. 20289639

[6] JO DL . Structural anatomy of the posterior pelvic compartment as it relates to rectocele. Am J Obstet Gynecol. 1999;180:815‐823. 10203649 10.1016/s0002-9378(99)70652-6

[7] Larson KA , Yousuf A , Lewicky‐Gaupp C , Fenner DE , DeLancey JO . Perineal body anatomy in living women: 3‐dimensional analysis using thin‐slice magnetic resonance imaging. Am J Obstet Gynecol. 2010;203:494.e15‐494.e21. 10.1016/j.ajog.2010.06.008 PMC3353407 21055513

[8] Maldonado PA , Carrick KS , Montoya TI , Corton MM . Posterior vaginal compartment anatomy: implications for surgical repair. Female Pelvic Med Reconstr Surg. 2020;26:751‐757. 30865031 10.1097/SPV.0000000000000707

[9] Nichols DH . Posterior colporrhaphy and perineorrhaphy: separate and distinct operations. Am J Obstet Gynecol. 1991;164:714‐721. 2003530 10.1016/0002-9378(91)90503-j

[10] Oberwalder M , Thaler K , Baig MK , et al. Anal ultrasound and endosonographic measurement of perineal body thickness: a new evaluation for fecal incontinence in females. Surg Endosc. 2004;18:650‐654. 15026922 10.1007/s00464-003-8138-5

[11] Santoro GA , Wieczorek AP , Stankiewicz A , Wozniak MM , Bogusiewicz M , Rechberger T . High‐resolution three‐dimensional endovaginal ultrasonography in the assessment of pelvic floor anatomy: a preliminary study. Int Urogynecol J Pelvic Floor Dysfunct. 2009;20:1213‐1222. 19533007 10.1007/s00192-009-0928-4

[12] Santoro GA , Shobeiri SA , Petros PP , Zapater P , Wieczorek AP . Perineal body anatomy seen by three‐dimensional endovaginal ultrasound of asymptomatic nulliparae. Color Dis. 2016;18:400‐409. 10.1111/codi.13119 26382090

[13] Asfour V , Digesu GA , Fernando R , Khullar V . Ultrasound imaging of the perineal body: a useful clinical tool. Int Urogynecol J. 2020;31:1197‐1202. 31828399 10.1007/s00192-019-04166-7 PMC7270988

[14] Zhou M , Shui W , Bai W , Wu X , Ying T . Ultrasonographic study of female perineal body and its supportive function on pelvic floor. Front Med. 2023;10:10. 10.3389/fmed.2023.1176360 PMC10410282 37564038

[15] Shobeiri SA , White D , Quiroz LH , Nihira MA . Anterior and posterior compartment 3D endovaginal ultrasound anatomy based on direct histologic comparison. Int Urogynecol J. 2012;23:1047‐1053. 22402641 10.1007/s00192-012-1721-3

[16] Shobeiri SA , Rostaminia G , White D , Quiroz LH . The determinants of minimal levator hiatus and their relationship to the puborectalis muscle and the levator plate. BJOG. 2013;120:205‐211. 23157458 10.1111/1471-0528.12055

[17] Trinh AT , Nippita TA , Dien TN , Morris JM , Roberts CL . Perineal length among Vietnamese women. Taiwan J Obstet Gynecol. 2017;56:613‐617. 29037545 10.1016/j.tjog.2017.08.006

[18] Lieming W , Baihua Z , Yingchun T , Yuyang G , Xian X . Morphological differences in the female anal sphincter complex between endoanal and exoanal ultrasound. Int Urogynecol J. 2023;34:545‐551. 36063193 10.1007/s00192-022-05341-z

[19] Shafik A , Sibai OE , Shafik AA , Shafik IA . A novel concept for the surgical anatomy of the perineal body. Dis Colon Rectum. 2007;50:2120‐2125. 17909903 10.1007/s10350-007-9064-8

[20] Mukaka MM . Statistics corner: a guide to appropriate use of correlation coefficient in medical research. Malawi Med J. 2012;24:69‐71. 23638278 PMC3576830

